# Attention Deficit Hyperactivity Disorder and Blood Lead Levels in Chinese Children

**DOI:** 10.1289/ehp.0900850

**Published:** 2009-07

**Authors:** Jack Brondum

**Affiliations:** Environmental Health and Epidemiology, Hennepin County Department of Human Services and Public Health, Hopkins, Minnesota, E-mail: jack.brondum@co.hennepin.mn.us

In their study of the relationship of blood lead levels (BLLs) in children 4–12 years of age and attention deficit hyperactivity disorder (ADHD), Wang et al. (2008) considered potential confounding variables and covariates with considerable thoroughness. For certain of these (e.g., low birth weight), the proportion of affected children generally accords well with reported prevalence in other settings [UNICEF and World Health Organization (WHO) 2004]. However, this was not so with case children with a family history of ADHD described by Wang et al. (2008). Only 21 (3.3%) of the 630 cases in their study had such a history.

ADHD and other externalizing disorders (e.g., conduct disorder) are known to have substantial genetic components, and ADHD heritability has been estimated to be 75% (Biederman and Faraone 2005; Gelhorn et al. 2006). Among children with ADHD or earlier definitions of the disorder, the reported proportions with at least one affected parent or sibling range from 9% to 64% (Biederman 2005; Biederman et al. 1990, 2008; Milberger et al. 1998; Roizen et al. 1996; Schachar and Wachsmuth 1990). This substantial body of work suggests a figure of 20–25% as a reasonable estimate of the proportion of first-degree relatives afflicted with ADHD, or 6–8 times that reported by Wang et al. (2008) in the families of their case children.

Wang et al. (2008) assessed family history of ADHD by psychiatric diagnoses noted in clinical reports. It is possible that such information was not systematically acquired in previous years and is thus under represented in these reports. This would actually be likely if ADHD was less well-defined or considered less often as a diagnosis in Anhui Province, China, when the parents of the 4- to 12-year-old children included in this study were of similar age. It is also possible that ADHD in this setting differed in some way from ADHD in other settings, although the rigorous diagnostic criteria used by the authors make this explanation less plausible.

In their backward stepwise logistic model (their Table 3), Wang et al. (2008) showed that ADHD in the child is positively associated with family history of ADHD and BLL (≥ 10 μg/dL vs. ≤ 5 μg/dL and 5–10 μg/dL vs. ≤ 5 μg/dL) and inversely associated with maternal education. Assuming that the reported odds ratio of 5.65 for family history of ADHD remained unaltered, a 6- to 8-fold increase in the number of case children with this exposure would commensurately increase the relevant Wald statistic and almost certainly reduce the Wald statistics of the associations with BLL and/or maternal education, conceivably to nonsignificant levels.

Wang et al. (2008) stated that their results reinforce findings from two previous studies of the relationship of BLL to ADHD, but it is unclear how this is so (Braun et al. 2006; Nigg et al. 2008). In the study by Braun et al. (2006), the cut-point of the highest BLL exposure quintile, the only one associated positively and significantly with ADHD, was 2 μg/dL; in the study by Nigg et al. (2008), the mean BLL for the ADHD-combined group was 1.26 μg/dL. Yet the mean BLL in control children studied by Wang et al.—by definition, ADHD-free—was 5.76 μg/dL, nearly 3 times the level reported by Braun et al. and 5 times that of Nigg et al. In neither of the earlier studies did the researchers adjust the BLL–ADHD relationship for family history of ADHD.

Familial transmission of ADHD and its diagnostic forebears has been documented for more than three decades (Cantwell 1972), and systematic assessment of the contribution of familial inheritance has been under way for more than two decades (Biederman 1986). Studying risk factors for ADHD with incomplete or no control of family history of ADHD is like studying risk factors for lung cancer with inadequate control of smoking history (Stevens and Moolgavkar 1984). Doing so may answer some questions, but the main question remains unanswered.

## Editor’s note

In accordance with journal policy, Wang et al. were asked whether they wanted to respond to this letter, but they chose not to do so.
